# Long Term Effects of Aversive Reinforcement on Colour Discrimination Learning in Free-Flying Bumblebees

**DOI:** 10.1371/journal.pone.0071551

**Published:** 2013-08-12

**Authors:** Miguel A. Rodríguez-Gironés, Alejandro Trillo, Guadalupe Corcobado

**Affiliations:** Department of Functional and Evolutionary Ecology, Estación Experimental de Zonas Áridas (EEZA-CSIC), Almería, Spain; Royal Holloway University of London, United Kingdom

## Abstract

The results of behavioural experiments provide important information about the structure and information-processing abilities of the visual system. Nevertheless, if we want to infer from behavioural data how the visual system operates, it is important to know how different learning protocols affect performance and to devise protocols that minimise noise in the response of experimental subjects. The purpose of this work was to investigate how reinforcement schedule and individual variability affect the learning process in a colour discrimination task. Free-flying bumblebees were trained to discriminate between two perceptually similar colours. The target colour was associated with sucrose solution, and the distractor could be associated with water or quinine solution throughout the experiment, or with one substance during the first half of the experiment and the other during the second half. Both acquisition and final performance of the discrimination task (measured as proportion of correct choices) were determined by the choice of reinforcer during the first half of the experiment: regardless of whether bees were trained with water or quinine during the second half of the experiment, bees trained with quinine during the first half learned the task faster and performed better during the whole experiment. Our results confirm that the choice of stimuli used during training affects the rate at which colour discrimination tasks are acquired and show that early contact with a strongly aversive stimulus can be sufficient to maintain high levels of attention during several hours. On the other hand, bees which took more time to decide on which flower to alight were more likely to make correct choices than bees which made fast decisions. This result supports the existence of a trade-off between foraging speed and accuracy, and highlights the importance of measuring choice latencies during behavioural experiments focusing on cognitive abilities.

## Introduction

Ever since the pioneering research of Karl von Frisch [1], bees stand among the most productive model systems in vision research [2], [3]. Until the 1990’s, behavioural data were used to infer the properties of the underlying neural mechanisms responsible for visual perception and information processing (see e.g. [4], [5]), a research approach known as reverse engineering. Some of these hypothesised properties were also investigated at the anatomical or neurophysiological levels [6], [7]. Although the focus of visual learning research has largely shifted towards the cognitive abilities of bees [2], [3], [8], [9], the debate around the mechanisms allowing insects to perceive and discriminate colours has not been settled and reverse engineering remains a valid strategy. The process of reverse engineering, however, is rendered more difficult by behavioural noise, which decreases the correlation between performance in experimental setups and perceptual constraints. Thus, although the results of behavioural experiments inform us of some capabilities that the visual system must have, other capabilities of the visual system may remain masked behind lack of motivation and other factors increasing behavioural noise [9]. Hence, for example, if in an experiment with proper controls bees learn to search for food in flowers of one particular colour, ignoring flowers of a different colour that have no food, we can conclude that their visual system allows bees to discriminate between the two colours. However, the opposite scenario, i.e. the finding that bees fail to choose one visual stimulus more often than another in an experiment, does not necessarily mean that they cannot perceive the difference between the stimuli – they may simply lack the motivation to choose it [10]. Consequently, if we are to use behavioural experiments to learn how visual information is acquired and processed, it is of paramount importance to devise experimental protocols that minimise noise.

Previous work has shown that the experimental protocol affects visual performance in behavioural tests. Thus, the performance of honeybees, *Apis mellifera*, and bumblebees, *Bombus terrestris*, in colour discrimination tasks increases if differential, rather than absolute conditioning is used during training [11], [12]. In differential conditioning the two colours are presented during training. Bees are trained to associate a target colour (rewarded conditioned stimulus, CS+) with nectar (positive unconditioned stimulus, US+) and the distractor (non-rewarded conditioned stimulus, CS-) with the absence of reward. After training, bees are asked to discriminate between the target and distractor colours. In absolute conditioning, on the other hand, bees are trained to associate a target colour with nectar and then, during the behavioural test, they are asked to discriminate between the target and a distractor colour, with which they have no prior experience. Performance improves further if the distractor colour is paired with quinine solution (negative unconditioned stimulus, US-), rather than with the mere absence of reward [10], [13]. Additionally, it has been suggested that the effect of differential conditioning and aversive reinforcement on performance could be mediated by an increase in attention [10], [12]. Differential conditioning, however, has not always been found to have a positive effect on colour discrimination by free-flying bees. Thus, Backhaus and co-workers [14] only found a weak difference, not statistically significant, between the performance of bees trained with absolute and differential conditioning. Likewise, while quinine solution is an effective aversive stimulus for free-flying bees [10], [11], [13], [15], in the laboratory constrained bees readily imbibe it and there is no evidence that quinine is aversive to constrained bees [16], [17]. In these laboratory studies, bees are harnessed to a metal structure and are not free to move. Furthermore, few studies specifically compare the effects of water and quinine on the learning process and consequent performance in visual tasks.

The schedule of reinforcement can affect learning directly, if it determines the strength of the associative connections formed in the brain, and indirectly, if it affects the internal state of individual (i.e. attentional and motivational processes). For instance, there is increasing evidence that, in colour discrimination tasks, the probability that a bee makes a correct decision increases with the time they invest in making the choice [13], [18], [19], and the unconditioned stimulus used during training can affect the time that bees invest in making a choice [13]. The purpose of this work was therefore to investigate the generality of the finding that using quinine solution as an aversive reinforcer enhances learning and final performance in bumblebees, and the extent to which such enhancement was mediated through changes in attention and motivation. Specifically, we asked whether early experience with a neutral/aversive US could affect decision times and learning rates after the nature of the reinforcer associated with the distractor stimulus was modified. To answer this question we used differential conditioning to train four groups of bees to discriminate between a target and a distractor colour. During two consecutive phases, we used water as a neutral US- and/or quinine as an aversive US- associated to the distractor colour. Depending on the US- used during each phase of the experiment, the four experimental groups were: Water-Water, Water-Quinine, Quinine-Water and Quinine-Quinine. Because quinine does not improve visual discrimination of perceptually dissimilar colours [10], only perceptually similar colours were used for the experiment. To investigate whether the effect of aversive reinforcement on visual learning is mediated through motivational processes, we must evaluate whether the effect of quinine is independent of the training stage at which it is presented. It is therefore important to have different groups of bees, experiencing water or quinine solution as reinforcer at different stages. This is the main difference between our work and previous studies, in which all bees have experienced the same sequence of reinforcers [13].

## Materials and Methods

### General Settings

Experiments were conducted indoors with bumblebees (*Bombus terrestris*) housed in a single-chamber nesting box (30×20×25 cm) connected via a Plexiglass tube to a flight arena (70×70×35 cm), with floors and walls lined with grey cardboard (Canson Mi-Teintes ref. 431). The flight arena was illuminated with two Philips TL-D90 Graphica 36w/965 white light tubes and one Philips TL-D 36w BLB UV light tube, 75 cm above its floor. Tube flicker was converted to 1,200 Hz. To obtain homogeneous illumination, the flight cage was covered by a wire mosquito net and illumination was diffused by one sheet of Rosco 216 UV-transmitting white diffusion screen (Rosco, Germany).

Bees had *ad libitum* access to pollen within their nest box. Outside experimental sessions the nest box was permanently connected to the flight arena, where bees were allowed to collect 20% (w/w) sucrose solution from randomly distributed artificial flowers (transparent Plexiglass cubes: 4×4×4 cm). The number of bees collecting nectar between sessions was highly variable, ranging between 5 and 25 for large colonies. All tested bees were individually marked with number tags. During sessions, only the experimental subject was allowed in the flight arena.

### Stimuli

**Figure 1:**
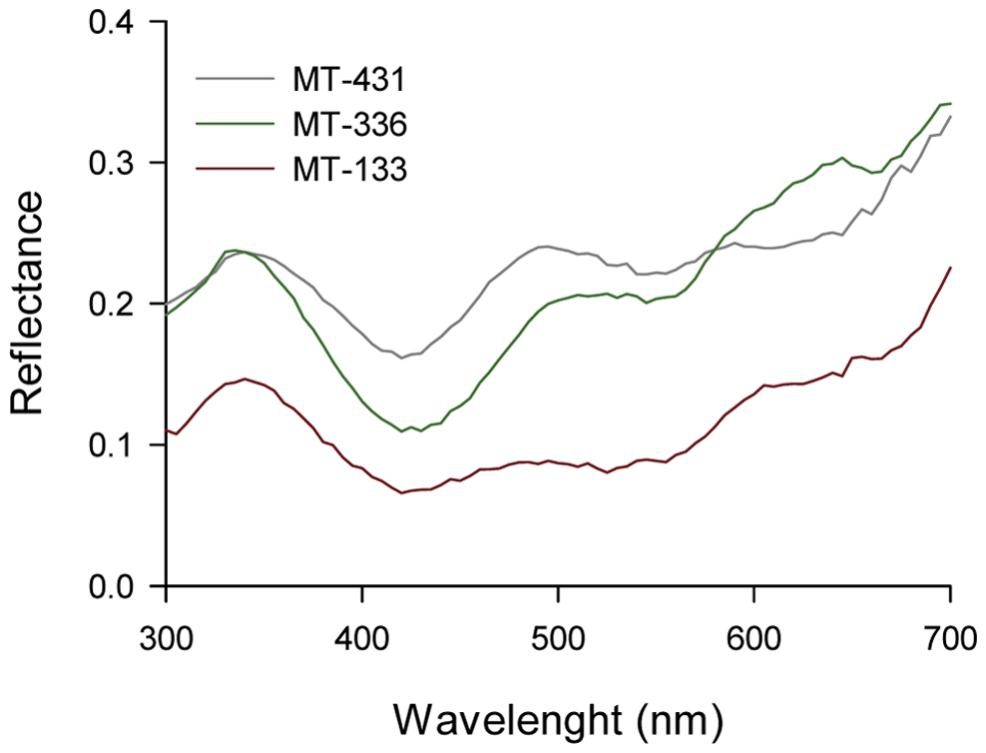
Spectral reflectance curves of the stimuli and background. Spectral reflectance of the grey background (Canson MT-431) and the two coloured stimuli (MT-336 and MT-133) in the 300–700 nm range.

Artificial flowers were set on top of cardboard squares (7×7 cm), cut from coloured papers (Canson Mi-Teintes refs. 133 and 336, which appeared reddish brown and greenish brown, respectively, to the human observer). One colour, the CS+, was used for target flowers and the other, the CS-, for distractor flowers. Each colour was used as CS+ for half of the bees and CS- for the other half within each experimental group. The spectral properties of incident light, as well as the reflectance spectra of the grey background and brown colour stimuli (Fig. 1), were measured with an Ocean Optics USB4000 spectrophotometer using a fibre-optic probe connected to a black probe holder to exclude ambient light at an angle of 45° to the surfaces measured. The spectrophotometer was connected to a PX-2 light source and attached to a PC running Ocean Optics Spectra Suite software. Reflectance data (300–700 nm) were generated relative to a white standard (Ocean Optics WS-1). For each sample, 10 spectra were averaged to reduce noise from the spectrophotometer with an integration time of 250 ms. We took three samples of each colour and averaged them to calculate photoreceptor excitation values and the loci of stimuli in the Backhaus colour-opponent model (COC) [4] and the Chittka hexagon model [20]. The two stimuli were close in the bee’s perceptual colour space: the chromatic distance between them was 0.94 COC units in the colour opponent colour space and 0.055 hexagon units in the hexagon colour space.

### Treatments and Training Procedure

**Figure 2:**
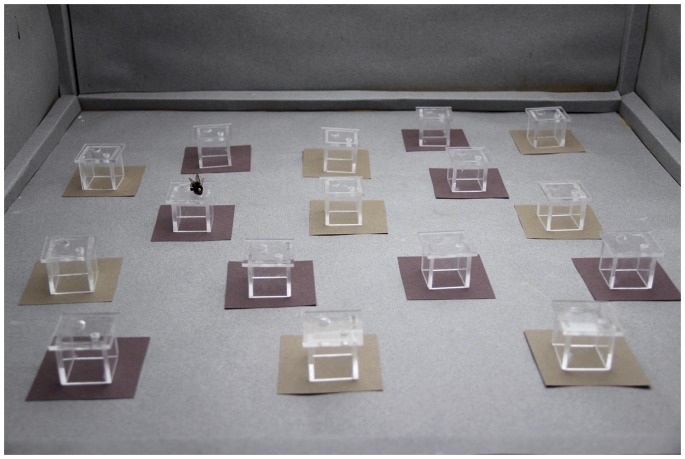
Flight arena used in this study. Photograph showing how stimuli were presented in association with the artificial flowers and distributed throughout the flight arena, as well as a marked bumblebee (marked with a numbered yellow tag) visiting a flower.

Forty bees were allocated to four possible treatments, 10 bees per treatment, in pseudo-random order to avoid correlations between time and treatment: in each group of four consecutive bees, one bee was randomly assigned to each treatment. Upon entering the flight arena during experimental sessions, individually marked bumblebees found eight target and eight distractor artificial flowers haphazardly distributed throughout the arena (Fig. 2). In each foraging trip (i.e. series of events taking place since the bee entered the flight arena until she returned to the nest), hereafter referred to as a trial, bees visited several flowers and returned to their nest after collecting the sucrose solution (US+) from 2–4 target flowers – the volume of reward per flower was adjusted during pretraining (in colourless flowers) so that bees typically consumed the nectar of three flowers before returning to their nest. Between trials, flowers were cleaned with 30% ethanol to remove olfactory cues and positioned in re-randomised locations.

**Figure 3:**
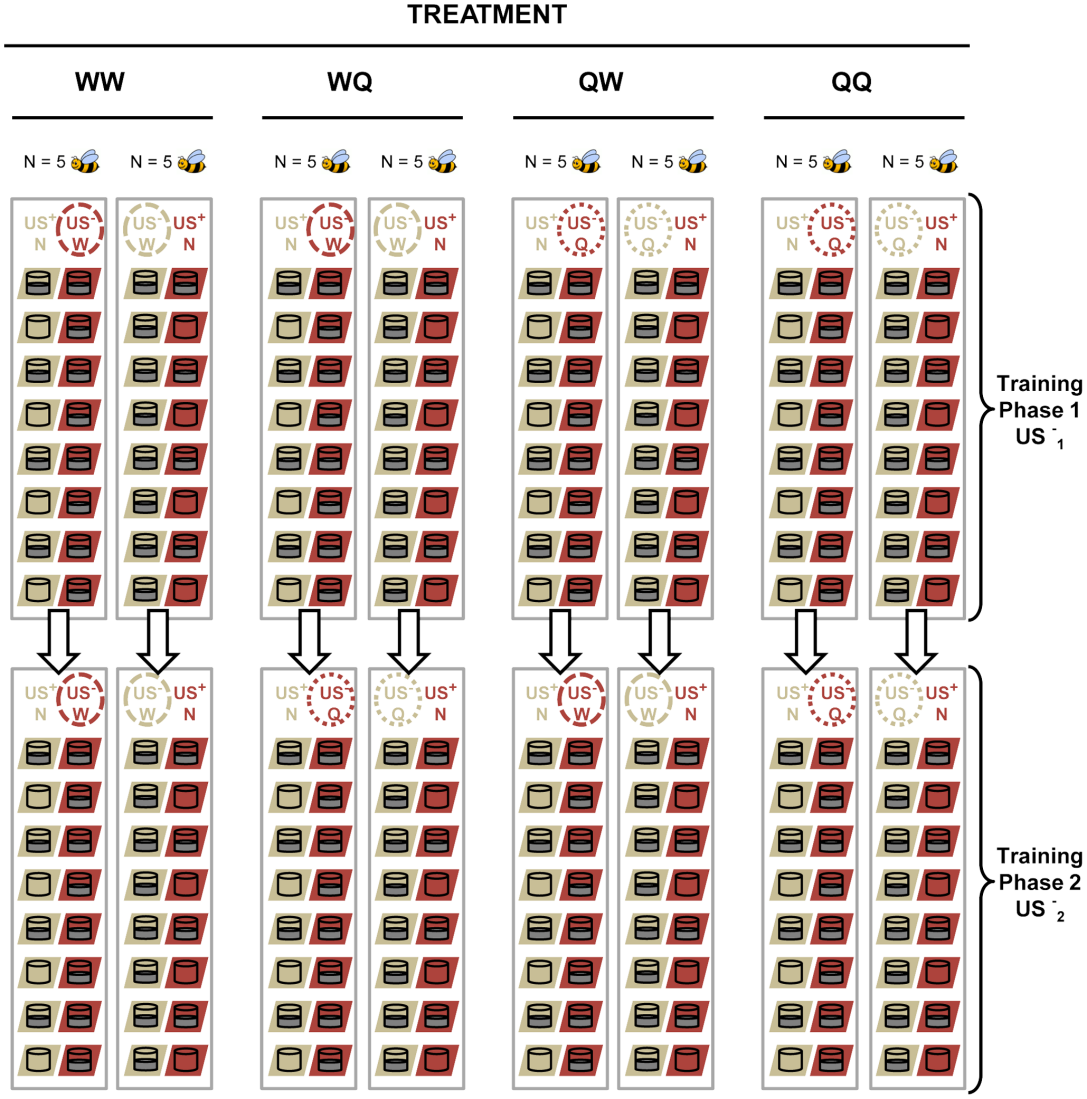
Experimental design. Combinations of unconditioned (US) and conditioned (CS) stimuli experienced by the bees during training. Each rectangular box indicates the contents of the flight arena at the beginning of each trial, for bees in the different treatments (represented by columns) in each experimental phase (top and bottom rows for phases 1 and 2, respectively). We studied the effect on learning of four reinforcement schedules, characterized by different choices of the US-. The US- could be water (W) or quinine (Q) during the whole experiment, or change from one to the other halfway through the experiment, accounting for the four experimental treatments: WW, WQ, QW and QQ. Regardless of the treatment, bees entering the arena encountered eight distractor flowers (US- column in each rectangular box), each one containing ca. 25 µl of the US- (represented by “filled cups”), and eight target flowers (US+ column). Four target flowers were empty (empty cups) and the other four contained ca. 25 µl of the US+ (sucrose solution – filled cups). Note that, in the experiment, distractor and target flowers were haphazardly distributed throughout the arena. Distractor and target flowers were identified by the colour squares (CS+ and CS-) on which they were set (represented by the cream and reddish parallelograms in the figure). The squares were cut from Canson Mi-Teintes cardboard (refs. 133 and 336). Forty bees were allocated to the different treatments, 10 bees per treatment. Within each experimental group, colour #133 was the CS+ for five of the bees and the CS- for the other five.

Each bee experienced 30 trials over a period of three hours (mean duration of the experiment ± s.e.m. = 187±7 minutes). Training took place during trials 1–14 (phase 1) and 16–29 (phase 2), and bees were tested in trials 15 and 30. Distractor flowers, CS-, contained US-_1_ during phase 1 and US-_2_ during phase 2. US-_1_ and US-_2_ could be either distilled water (W) or 0.12 M quinine hydrochloride dihydrate solution (Q), in a full factorial design. Thus, the four experimental groups of bees were characterised by the combination US-_1_/US-_2_, as follows: WW, WQ, QW and QQ (Fig. 3).

Irrespective of treatment, the target colour stimulus (CS+) was paired with 60% (w/w) sucrose solution with a 0.5 probability (in each trial, four out of eight target flowers were rewarded, the other four were empty), while all eight distractor flowers were paired with the US-. We chose to reward only half of the CS+ flowers (partial reinforcement schedule) because we planned to test bee performance after training in the absence of rewarded flowers (i.e., during extinction – trials 15 and 30) and partial reinforcement schedules typically lead to more robust performance in extinction [21]. Most bees, however, behaved markedly differently during training trials and extinction tests, often approaching flowers without landing during the tests and making it difficult to unambiguously assess choices (there was little between-observer consistency in the tests). For this reason, the results of the extinction tests will not be reported.

To measure the accuracy of individual foraging strategies, and its progress as a result of learning, we recorded the number of target and distractor flowers on which bees landed in each trial, noting for each visit to distractor flowers whether bees contacted the US- with their proboscis (hereafter referred to as drinking opportunities). Because we could not always detect whether bees actually drank from the US-, the number of drinking opportunities must be seen as an upper bound to the number of US- ingestions. To measure the time that bees spent making decisions we videotaped trials 14 and 29 (last trials of the phases 1 and 2, respectively). A frame-to-frame analysis of the recordings provided, for each bee, the median time elapsed since the bee left a flower until it landed on the following one. We refer to this time as choice latency.

### Remote Detection of Quinine Solution

Honeybees are unable to discriminate sucrose solution and quinine remotely by olfactory cues [10]. To confirm that bumblebees could not use olfactory cues in the discrimination of target and distractor flowers, we trained five additional bees to forage at colourless flowers. After learning to exploit these flowers, each bee experienced 20 trials in which the arena contained eight flowers with 25 µl sucrose solution and eight flowers with 25 µl quinine solution. Flowers were visually identical, and their spatial position was re-randomised in each trial. We recorded the number of quinine and sucrose flowers that bees visited per trial: if bumblebees could discriminate sucrose solution and quinine remotely by olfactory cues, the proportion of sucrose flowers visited would be greater than 0.5.

### Statistical Analyses

To investigate the effect of reinforcement schedule on visual learning by free-flying bumblebees, we looked at the interrelationship between US-, choice latency, acquisition and final performance in the discrimination task (i.e. changes in the proportion of correct choices during the successive training trials –acquisition- and proportion of correct choices at the end of each of the two experimental phases -final performance).

#### Choice latency

We used a general linear model (GLM) to evaluate the within-individual consistency of choice latencies and their dependence on the US-. Specifically, we performed a GLM with choice latency during trial 29 as a dependent variable, the choice of US-_1_ and US-_2_ as fixed factors (full factorial design), and the choice latency during trial 14 as a covariate. Because choice latency in trial 29 was highly correlated with choice latency in trial 14 (see results), we used the average of these two measures in subsequent analyses where choice latency was included as a covariate –the average is less noisy than either measure alone.

#### Acquisition of the discrimination task

We pooled the 28 training trials in six blocks (trials 1–5, 6–10, 11–14, 16–20, 21–25 and 26–29) and calculated, for each bee, the proportion of correct choices in each block of trials. The effect of US- on learning rate was studied with repeated-measures analyses of variance (ANOVA) on the correct choices over the three blocks of an experimental phase, having treatment as a fixed factor and choice latency as a covariate. For phase 1, the dependent variable (within-individual repeated measures) was the proportion of correct choices during blocks 1–3, the US-_1_ was used as a fixed factor and choice latency as a covariate. For phase 2, we analysed the effect of US-_1_, US-_2_ and their interaction on the proportion of correct choices during blocks 4–6, with choice latency as a covariate. The interactions between block and treatment and block and choice latency were included in both analyses.

#### Final performance

Due to our inability to unambiguously assign flower choices during the extinction tests (low inter-observer repeatability), we used the proportion of correct choices during the last block of each experimental phase as a proxy for final performance. The proportion of correct choices (blocks 3 and 6 for phases 1 and 2, respectively) was analysed with a GLM having treatment (US-_1_ for block 3; US-_1_, US-_2_ and their interaction for block 6) as a fixed factor and average choice latency as a covariate. While this analysis focuses on the ability of bees to discriminate between target and distractor flowers at the end of training, the previous analysis (acquisition) investigates the rate at which discrimination ability was acquired.

#### Ingestion of US-

To study how the maximum number of US- ingestions changed through time as a function of treatment, we performed generalised linear models with Poisson distributions and logarithmic link functions on the number of drinking opportunities at distractor flowers, having treatment (US-_1_ for phase 1; US-_1_, US-_2_ and their interaction for phase 2) as fixed factor(s). For these analyses, we determined statistical significance from type II log-likelihood ratio tests.

#### Remote discrimination

In the experiment without colour stimuli, to determine whether bees could discriminate sucrose and quinine solution remotely by olfactory cues we performed binomial tests on the number of sucrose and quinine flowers visited by each bee over the last five trials (trials 16–20). If the probabilities of selecting sucrose flowers were greater than 0.5, the data would provide evidence of remote discrimination.

All analyses were performed with Statistica v. 10.

## Results

### Consistency of Individual Foraging Strategies

**Figure 4:**
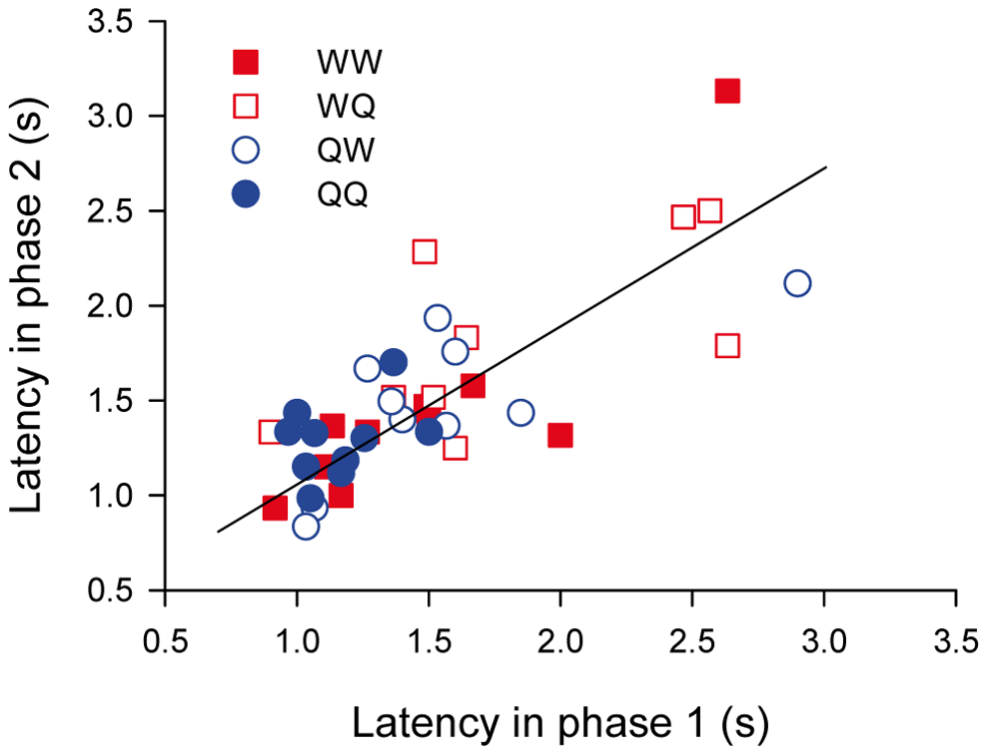
Correlation between choice latencies at the end of phases 1 and 2. Each dot represents the choice latencies (in seconds) during trials 14 and 29 for an individual bee. Symbol type indicates the treatment to which bees were allocated: red squares represent bees with US-_1_ = W, blue circles represent US-_1_ = Q. Filled symbols correspond to bees which had the same reinforcer in phases 1 and 2 of the experiment, and empty symbols to bees which had different reinforcers.

Bumblebees and honeybees are known to face a trade-off between increasing the speed at which they solve colour-discrimination tasks and the accuracy of their choices [13], [18], [19], and individual bees have been shown to be consistent in their choice of foraging strategy within this speed-accuracy gradient [13], [19]. Our results confirmed the consistency of the individual foraging strategies despite changes in the choice of US-: bumblebees which made fast decisions at the end of phase 1 continued making fast decisions at the end of phase 2 independently of the treatment. Indeed, choice latencies at the end of phase 2 (trial 29) were highly correlated with choice latencies at the end of phase 1 (trial 14; F_1,35_ = 39.87, p<0.000001– Fig. 4). Bees, however, spent as much time inspecting flowers prior to landing regardless of whether distractor flowers contained water or quinine: choice latencies at the end of the experiment were not affected by the choice of US- during phase 1 (F_1,35_ = 0.50, p = 0.48), during phase 2 (F_1,35_ = 1.37, p = 0.25) or their interaction (F_1,35_ = 0.23, p = 0.63). As we will see below, choice latency was strongly correlated with performance. Therefore, and in agreement with previous studies, individuals were consistent in their choice of foraging strategy within the continuum from fast-inaccurate to slow-accurate.

### Acquisition of the Discrimination Task

**Figure 5:**
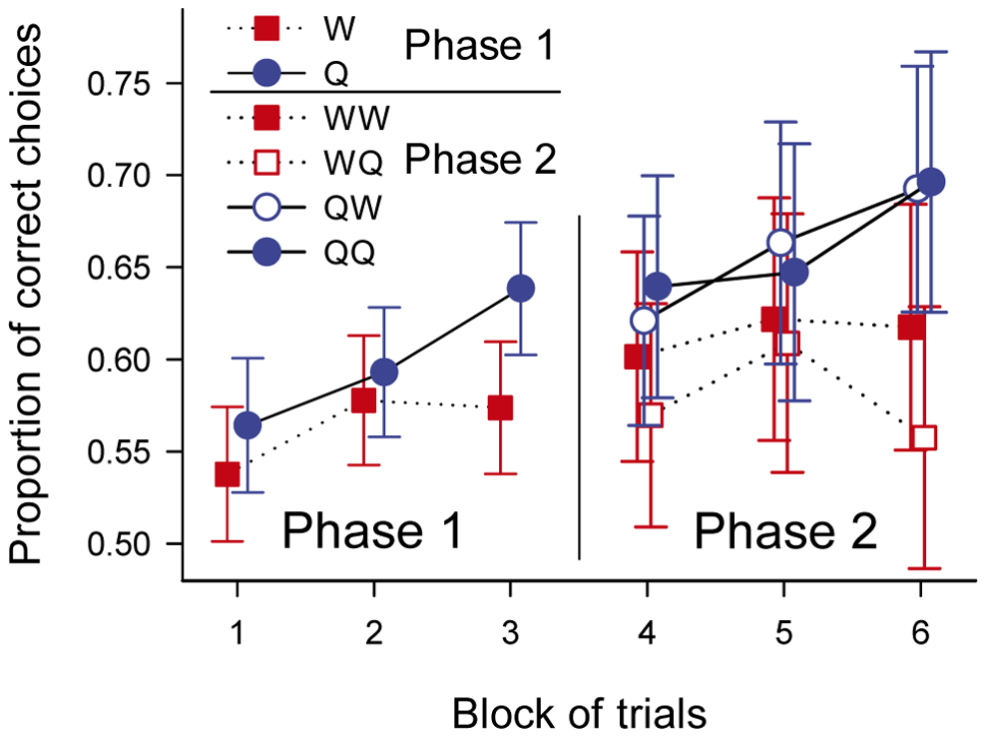
Learning performance. Average proportion of correct choices (estimated for the mean choice latency) over a block for bees which had water (red squares) and quinine (blue circles) as the negative reinforcer (US-_1_) during phase 1. For blocks 4–6, filled symbols correspond to bees which had the same reinforcer in phases 1 and 2 of the experiment, and empty symbols to bees which had different reinforcers. Error bars denote 95% confidence intervals.

Phase 1 was divided in three blocks of trials. The proportion of correct choices increased steadily from block to block for bees trained with quinine solution as US-_1_, but reached a plateau after the second block when distractor flowers offered water (Fig. 5). Nevertheless, this increase in the proportion of correct choices over time was not statistically significant (block of trials: F_2,74_ = 0.72, p = 0.49). Besides, the choice of aversive reinforcer during phase 1 (US-_1_: F_1,37_ = 2.88, p = 0.098) and its interaction with block of trials (block·US-_1_: F_2,74_ = 2.07, p = 0.13) had no-significant statistical effects on performance during phase 1, although bumblebees trained with quinine tended to perform better than bumblebees trained with water (Fig. 5).

**Figure 6:**
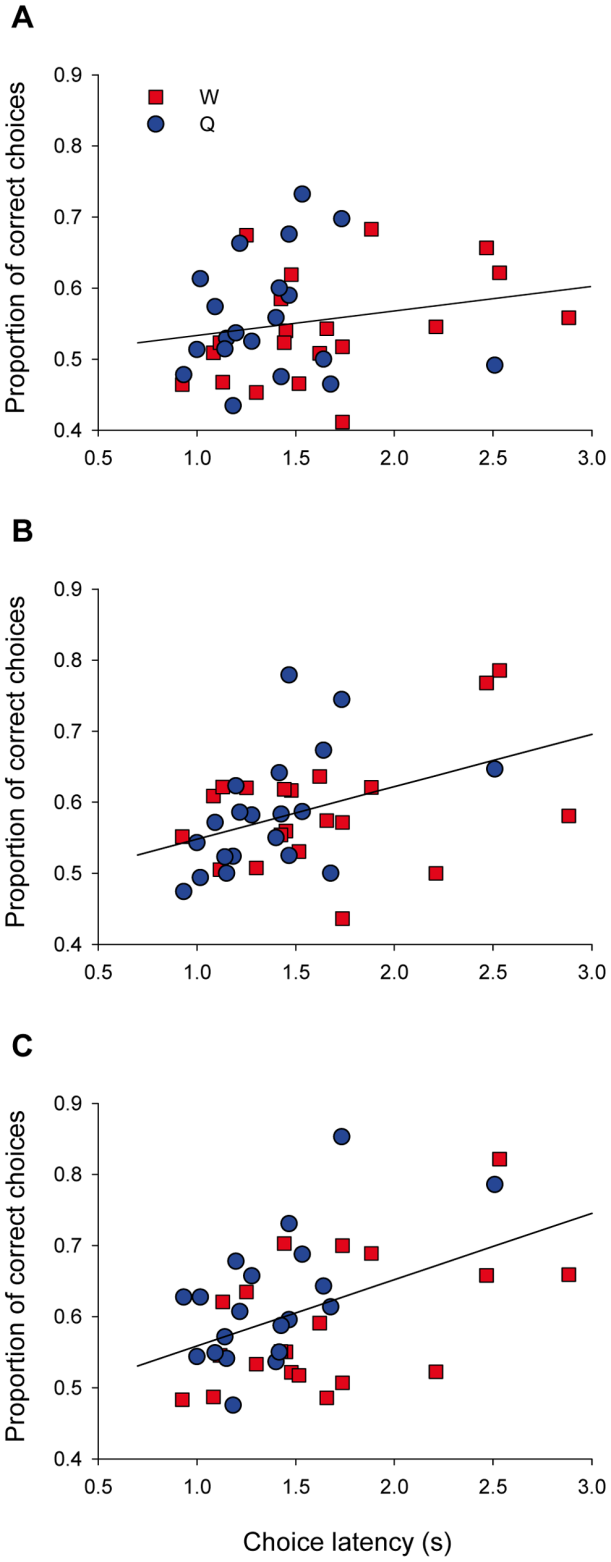
Relationship between choice latency and performance during phase 1. Each dot represents the choice latency and performance for an individual bee. Latencies are calculated as the averages of those measured in trials 14 and 29. Performance is the proportion of correct choices over a block, for (A) block 1 (trials 1–5), (B) block 2 (trials 6–10) and (C) block 3 (trials 11–14). Symbol type indicates whether bees experienced water (red squares) or quinine (blue circles) as negative reinforcer during phase 1. Note the increase in the slope of the regression line as we move from A to C.

Throughout phase 1, there was a positive relationship between choice latency and accuracy: bumblebees that spent longer times choosing the next flower to visit were more likely to visit target flowers (choice latency: F_1,37_ = 11.82, p = 0.001). However, the relationship between choice latency and proportion of correct choices changed throughout phase 1 (Fig. 6), as evidenced by a significant effect of the interaction between block and choice latency on the proportion of correct choices (block·choice latency: F_2,74_ = 3.23, P = 0.045). Early on (block 1), there was little effect of choice latency on the proportion of correct choices (mean ± standard error of slope 0.04±0.03, t = 1.52, p = 0.14: Fig. 6A), but the slope of the relationship increased during the second (slope 0.08±0.03, t = 2.88, p = 0.007: Fig. 6B) and third (slope 0.11±0.03, t = 4.06, p = 0.0002: Fig. 6C) blocks.

During phase 2 (blocks 4 to 6), the rate at which the proportion of correct choices increased from block to block was greater for bees which had been trained with quinine as US- during phase 1 than for bees trained with water during phase 1 (block·US-_1_: F_2,70_ = 3.24, p = 0.045). Besides, the overall proportion of correct choices during phase 2 was higher for bees trained with quinine during phase 1 (US-_1_: F_1,35_ = 5.16, p = 0.029: Fig. 5). Surprisingly, the choice of negative reinforcer during phase 2 had no effect on learning rate: we found no effect of US-_2_ neither on the overall proportion of correct choices during this phase (US-_2_: F_1,35_ = 0.38, p = 0.54), or on the rate at which the proportion of correct choices changed from block to block (block·US-_2_: F_2,70_ = 0.30, p = 0.74). Furthermore, the proportion of correct choices during phase 2 was unaffected by the interaction between the choice of negative reinforcer during phases 1 and 2 (US-_1_·US-_2_: F_1,35_ = 0.42, p = 0.52) or the triple interaction between block of trials, US-_1_ and US-_2_ (block·US-_1_·US-_2_: F_2,70_ = 0.70, p = 0.50). However, the positive relationship between choice latencies and proportion of correct choices persisted during phase 2 (choice latency: F_1,35_ = 8.21, p = 0.007), although this time the relationship no longer changed with prolonged training (block by choice latency interaction: F_2,70_ = 1.87, p = 0.16).

To summarise, only choice latencies and the choice of negative reinforcer during the first phase of the experiment had an effect on the acquisition of colour discrimination abilities. The use of quinine solution as aversive reinforcer during the initial phase of the experiment enhanced the acquisition of the discrimination task, regardless of the aversive reinforcer used during the second phase of the experiment. Moreover, bees which spent a prolonged time before choosing on which flower to land were more likely to select target, as opposed to distractor, flowers.

### Final Performance

The effects of US-_1_ and choice latencies on the acquisition of the discrimination task analysed in the previous heading resulted in predictable effects on performance at the end of training. At the end of phase 1 (block 3), bees which had been trained with quinine made a higher proportion of correct choices than bees trained with water during phase 1 (US-_1_: F_1,37_ = 6.37, p = 0.016), and longer choice latencies lead to greater proportions of correct choices (choice latency: F_1,37_ = 96.79, p<0. 0001). The same factors determined the proportion of correct choices at the end of phase 2 (block 6). Specifically, after controlling for the positive effect of choice latency (choice latency: F_1,35_ = 10.51, p = 0.003), the proportion of correct responses in block 6 was affected by the choice of negative reinforcer during phase 1 (US-_1_: F_1,35_ = 9.54, p = 0.004), but not by the choice of negative reinforcer during phase 2 (US-_2_: F_1,35_ = 0.73, p = 0.40) or their interaction (US-_1_·US-_2_: F_1,35_ = 0.82, p = 0.37).

### Ingestion of US-

Bees spent little time at distractor flowers, and never consumed the ca. 25 µl of US- that they offered. After excluding a bee from the WW group which had 52 US- drinking opportunities during phase 1 (and none in phase 2), the number (mean ± standard error) of drinking opportunities at distractor flowers during phase 1 was 1.05±0.22 when water was used as US-_1_ and 2.75±0.45 when quinine solution was used as US-_1_. This difference was statistically significant (US-_1_: χ^2^ = 15.24, d.f. = 1, p<0.0001). Most US- drinking opportunities were concentrated on the first few trials – in phase 1 we recorded only two drinking opportunities after the fifth trial. During phase 2, the number of US- drinking opportunities we recorded was low if US-_1_ = US-_2_ (0.22±0.15 and 0.1±0.1 for the WW and QQ groups, respectively) and similar to the number recorded during phase 1 otherwise (1.5±0.40 and 1.2±0.2 for the WQ and QW groups, respectively). The main effects of US-_1_ and US-_2_ were not statistically significant (χ^2^ = 0.l75, d.f. = 1, p>0.35), but the interaction was highly significant (χ^2^ = 20.86, d.f. = 1, p<0.0001). Once again, most US- drinking opportunities took place at the beginning of phase 2: we recorded only three drinking opportunities at distractor flowers in the last 10 trials of phase 2. Drinking opportunities at distractor flowers were therefore tightly linked to the novelty of the US-.

### Remote Detection of Quinine Solution

None of the bees learned to discriminate between colourless target and distractor flowers. Over the last five trials, the proportion of target flowers visited ranged from 0.43 to0.54 and never departed significantly from 0.5 (two-tailed binomial test, p>0.45 for all bees). It follows that, in our experimental setup, bumblebees were unable to discriminate sucrose and quinine solutions remotely by olfactory cues.

## Discussion

Our results confirm that the choice of reinforcer can affect the process of learning a colour-discrimination task by free-flying bees [10], [13]. More interestingly, with our setup the nature of the reinforcer at the beginning of the experiment determined the learning rate throughout its entire course, even when the nature of the reinforcer changed halfway through the experiment. We also confirmed that individual bees are consistent in their choice of foraging strategy within the continuum from fast-inaccurate to slow-accurate, although in our experiment choice latencies were unaffected by the nature of the reinforcer.

### Colour Discrimination vs. Achromatic Modulation of Long Wavelength Receptors

Bees use different neural pathways to solve different visual tasks. For instance, the colour channel implicated depends on whether bees detect motion cues or stationary targets [22], [23] and on the visual angle that the stimulus subtends [24]–[26]. Chromatic perception results from the combination, through opponent processing, of the information gathered by short, medium and long wavelength receptors [4], [27], [28] and should be distinguished from detection of differences in the response of photoreceptors of a single type. In particular, modulation of the response of long wavelength photoreceptors is involved in a number of visual tasks, such as detection of stimuli that subtend small visual angles [24]–[26] and detection of motion cues [22], [23]. Because the colour stimuli we used in the experiment differed in the excitation level that they produced on all three photoreceptor types, bees could, in principle, use colour discrimination or modulation of the long wavelength photoreceptor to select on which flowers to land. Nevertheless, given the size of our flowers, we should expect bees to base their decisions on colour signals in our experiment [24]–[26]. Moreover, it has recently been shown that free-flying honeybees can use colour discrimination, independently of long wavelength receptor modulation, to discriminate between perceptually similar colours [29].

### Aversive Value of Water and Quinine Solution

Before the start of the experiments, bees were trained to collect 20% sucrose solution from colourless flowers. Thus, when bees first landed on a distractor flower during phase 1, it seems likely that they directly drank the reward offered by the flower. Most bees in groups QW and QQ showed a strong aversive response the first time that they encountered quinine. They dashed away from the flower, entered a bout of frenzy activity and, occasionally, returned to their nest and refused to forage for extended periods of time. This response disappeared quickly, normally after as few as one or two visits to distractor flowers, and was never observed among bees encountering water. Therefore, drinking quinine solution has a stronger aversive value for free-flying bees than drinking water. Throughout most of the experiment, however, bees did not drink from the US-: all but a few US- drinking opportunities were concentrated in the first trials of phases 1 and 2 (and the observation of a drinking opportunity does not imply that the bee actually ingested the US-). It is possible that, after experiencing that not all flowers contained sucrose solution, bees inspected the contents of flowers prior to ingesting it. For instance, bees may have checked with their antennae the contents of the flowers, immediately departing from flowers without sucrose solution: although bees cannot detect quinine with their antennae [17] they can detect sucrose [30], and hence its absence. As a result, quinine solution probably had a strong aversive effect at the beginning of the experiment, but a neutral or mildly aversive effect later on. Finally, the scarcity of drinking opportunities associated with water, and their disappearance after the initial few trials, indicates that bees were not motivated to drink water. Water was, therefore, a neutral or mildly aversive stimulus.

### Effect of Quinine Solution on Learning

Previous studies have found that bees are more likely to learn a difficult colour discrimination task if the distractor stimulus, CS-, is paired with a noxious substance such as quinine than if it is paired with water [10], [13]. Our results agreed with these studies, up to a point. Specifically, we found that the rate of acquisition of the colour discrimination task was determined by the unconditioned stimulus paired with distractor flowers during the first phase of the experiment, and was not affected by the unconditioned stimulus used during phase 2. Despite this caveat, it seems clear that data from discrimination experiments in which the CS- was not paired with an aversive stimulus are unlikely to reflect the limits of the visual system of bees.

If quinine solution has an immediate aversive effect at the level of gustatory receptors [10] but learning during phase 2 was determined by the choice of US-_1_, the effect of quinine on learning could not be mediated by the trial-by-trial aversive value of quinine. Instead, it must have resulted from a mid-term effect of early exposure to quinine. As we have seen, it seems likely that bees did not ingest water or quinine after the initial trials, and that both stimuli had, in the absence of ingestion, similar aversive values. Our results could be explained if ingestion of quinine by QW and QQ bees at the start of the experiment had an arousal effect. The hypothesis that visual learning is enhanced by general arousal can readily be tested. For example, bees could be divided in two groups, presented during pre-training with a mixture of colourless flowers containing nectar and water or nectar and quinine. According to the arousal hypothesis, bees exposed to quinine during pre-training should learn faster a subsequent colour discrimination task than bees exposed to water, even if both groups were trained with water (or quinine) as US-. Furthermore, the same effect should be obtained if another noxious stimulus was used during pre-training, or if the learning task involved a different domain, such as shape or odour discrimination.

Our results contrast with those reported by Chittka and colleagues [13] who used differential conditioning to train bumblebees to discriminate between two perceptually similar colours, in a setup relatively similar to ours. In their experiment, all bees first experienced water as US-, then quinine solution and finally water again. Following each training phase, individual bees were subject to a discrimination test. Performance was poor after the first phase with water as US-, increased when quinine solution was used as US-, and decreased again when water was once more used as US- in the third phase of the experiment [13]. The main difference between the two experiments lies in the duration of training phases (two days with water, one day with quinine and one final day with water in the Chittka et al. [13] experiment vs. 1.5 hours per phase in our experiment). It therefore appears that the arousal effect of the aversive stimulus determines the learning rate in the midterm (about three hours in our experiment), but disappears in the long term. There were, however, other differences between the two experiments. In the Chittka et al. [13] experiment, bees were rewarded every time they landed on target flowers and bees were trained in groups of five (Lars Chittka, personal communication), while in our setup only 50% of target flowers contained sucrose solution and only the experimental bee was allowed in the flight arena during training. While we believe that the differences between the two sets of results most likely stem from differences in the time course of the experiments, it is impossible to be certain of this without controlling for the other procedural differences. It should also be pointed out that different studies use different aversive substances. Although there is a tendency to use 0.12% quinine hemisulphate solution for experiments with *B. terrestris* [11], [13], [31] or 0.06 M quinine hydrochloride dihydrate for experiments with *A. mellifera* [10], [32], other combinations have also been reported, such as the use of 0.12% quinine hemisulphate solution for an experiment with *A. mellifera* [33], or 0.012% quinine hemisulphate solution for experiments with *A. mellifera* and *B. terrestris* [26]. To the best of our knowledge, there is no systematic comparison of the effect of one substance with the other at different concentrations.

### Individual Variability in Foraging Strategies

One of the clearest results of our study was that bees which took more time to choose made more accurate choices, and that bees were consistent in their choice of foraging strategy. This result has important conceptual and methodological implications and confirms a number of previous studies [13], [18], [19]. The existence of a trade-off between increasing foraging speed and accuracy provides information about the constraints to which the neural system of the bees is subject and underlies the need to search the neural structures in the bee brain responsible for this trade-off [34]. Within and between-colony variability in the choice of foraging strategy along the continuum from fast-inaccurate to slow-accurate may, in turn, have important ecological and evolutionary consequences [19], [35], [36]. Recent studies have also found consistent individual differences in learning ability. For instance, bumblebees which are good at discriminating colours are also good at discriminating shapes or odours [37]. These results could support the hypothesis that learning ability is independent of the domain in which it operates [38]. Nevertheless, across-task consistency in learning ability is also to be expected from individual consistency in the choice of decision strategy within the gradient from fast-inaccurate to slow-accurate: bees with long choice latencies are likely to make accurate choices regardless of whether they have to discriminate between two colours, shapes or odours. Likewise, individual consistency in choice latencies could help explain the finding that learning rate during a colour discrimination task is correlated with learning rate during reversal learning – when the colour associated with the reward is reversed – in free-flying bumblebees [39].

The effect of choice latency on performance is not restricted to free-flying bees facing visual tasks. Rather, it seems to be a general phenomenon, common to many cognitive domains and species. For instance, humans solving an interval-timing discrimination took longer to respond when the task was difficult, and were more likely to choose the correct response after long than after short choice latencies, a finding that has been linked to attentional processes [40].

### Methodological Implications

Partial-reinforcement schedules do not seem to enhance the robustness of bee behaviour during extinction tests. Rather, they appear to lead to erratic behaviour during these tests and are therefore to be avoided.

The aversive value of quinine solution may be, to some extent, under the behavioural control of bees. In particular, it seems likely that bees can use their antennae to reject water and quinine flowers without experiencing the aversive value of quinine. To tease apart the effects of attention and aversiveness of the US on learning, it is important to use an experimental protocol where bees cannot visit distractor flowers without experiencing the aversive value of their reinforcer. Enclosing the flower reward in such a way that bees must insert their proboscis through a narrow opening to access it may impede them from using antennal receptors. Alternatively, non-gustatory aversive stimuli – such as electric shocks – could be associated with distractor flowers.

Much of the variability in performance was explained by differences in choice latency (Fig. 6). Differences between treatments became statistically non-significant if the correction for choice latency was removed from the analyses. Likewise, in the study by Chittka et al. a fair share of the variance in performance was explained by variability of individual foraging strategies, and removing this covariate from analyses would probably render treatment non-significant [13]. In general, measuring choice latency allows us to control for it in statistical analyses and increases the probability of detecting existing differences between groups. For instance, the result that there was a small, non-significant difference, in the performance of honeybees trained with absolute and differential conditioning [14] might have become statistically significant if the authors had measured decision times and included them in their statistical model.
